# Continuous-time modeling of cell fate determination in Arabidopsis flowers

**DOI:** 10.1186/1752-0509-4-101

**Published:** 2010-07-22

**Authors:** Simon van Mourik, Aalt DJ van Dijk, Maarten de Gee, Richard GH Immink, Kerstin Kaufmann, Gerco C Angenent, Roeland CHJ van Ham, Jaap Molenaar

**Affiliations:** 1 Biometris, Plant Sciences Group, Wageningen University and Research Center, Wageningen, The Netherlands; 2 PRI Bioscience, Plant Research International, Wageningen University and Research Center, Wageningen, The Netherlands; 3 Netherlands Consortium for Systems Biology, Science Park 904, 1098 XH Amsterdam, The Netherlands

## Abstract

**Background:**

The genetic control of floral organ specification is currently being investigated by various approaches, both experimentally and through modeling. Models and simulations have mostly involved boolean or related methods, and so far a quantitative, continuous-time approach has not been explored.

**Results:**

We propose an ordinary differential equation (ODE) model that describes the gene expression dynamics of a gene regulatory network that controls floral organ formation in the model plant *Arabidopsis thaliana*. In this model, the dimerization of MADS-box transcription factors is incorporated explicitly. The unknown parameters are estimated from (known) experimental expression data. The model is validated by simulation studies of known mutant plants.

**Conclusions:**

The proposed model gives realistic predictions with respect to independent mutation data. A simulation study is carried out to predict the effects of a new type of mutation that has so far not been made in *Arabidopsis*, but that could be used as a severe test of the validity of the model. According to our predictions, the role of dimers is surprisingly important. Moreover, the functional loss of any dimer leads to one or more phenotypic alterations.

## Background

For various plant species, floral organ specification has been successfully linked to spatial gene expression patterns according to the well-known ABC model [1]. This model has recently been extended to five gene classes (ABCDE) to explain novel floral mutants and to accommodate functions that specify ovule development and the establishment of floral context [2-7]. Despite these modifications, however, the ABCDE-model remains a static, qualitative model that does not describe the detailed molecular interactions involved, nor the temporal and spatial gene expression patterns that these interactions induce.

To model the molecular interactions involved in floral organ formation, various approaches have been used, mostly in terms of boolean networks. A boolean network approach was successfully applied to recover known stable states and to predict the existence of unknown interactions, [8-10]. Also, a stochastic type of boolean network, and a differential equations model, that can be considered as a first approximation of kinetic-reaction equations, have been proposed [11]. These types of models are especially suited for qualitative analysis of large model structures. The validity of a candidate model can be tested by comparing the steady states of the model with those measured experimentally. In [12], a general review is given on the various modeling approaches applied to gene regulatory networks, ranging from basic logical models to very extensive stochastic simulation algorithms, and a review specifically on stochastic methods is given in [13]. In [14], a stochastic model of the autoregulatory loop of the B-type genes *PISTILLATA (PI) *and *APETALA (AP3) *in *Arabidopsis *and *Antirrhinum *is described.

Ordinary differential equations (ODE) have been used extensively to model gene regulatory networks, including the Notch signaling pathway [15], the Zebrafish Somitogenesis Clock [16], the carbon starvation response in E. coli [17], and the toggle-switch gene network [18]. This type of model allows a quantitative, continuous-time analysis. However, for quantitative reliability, detailed parameter information is essential. This information is often not available, and instead the parameters are estimated indirectly by an identification procedure. In [19] an overview is presented of ODE networks that have been identified or are suitable as benchmark test.

Recently, gene expression data sets have become available for the genes involved in specification of floral organ identity. In [20,21], time series of gene expression are presented for each class of genes in the ABCDE group. For most ABCDE genes, the majority of which are members of the MADS-box transcription factor family, the way in which they are activated is known from experiments [22,23]. Furthermore, it has been shown that MADS box transcription factors regulate their own and each other's expression via various autoregulatory loops (reviewed in [24,25]). These two information sources open the door for ODE model development. There is also considerable evidence that MADS proteins play a crucial role in the autoregulatory repression or activation of specific sets of target genes [25-28].

The consensus MADS domain protein target site (CArG-box) is a palindromic sequence and structural analysis of the SRF MADS domain region bound to DNA has revealed its binding in a dimeric form [29]. Based on the identification of higher-order complexes and the fact that more than two different MADS domain proteins are essential for specifying organ identity, it has been hypothesized that plant MADS transcriptional complexes consist of quartets assembled from two active dimers [30-33]. The structure of this paper is as follows. First, we develop an ODE model for MADS box gene expression. The role of dimers is explicitly incorporated in this model. Second, we show that this model is able to simulate the dynamics of experimental time series data. Third, after the model has been fitted to experimental data, the predictive power of the model is assessed by comparing model predictions of ABCDE gene mutants with independent mutant experiments. Finally, we use the model to predict the effects of changes in the topology of the underlying protein-interaction network. We conclude that the model has a good predictive power with respect to mutations. A simulation study of a new mutant type in which the formation of specific dimers is disrupted, shows that each dimer function is essential for proper organ formation.

### The ABC model for Arabidopsis

**Figure 1:**
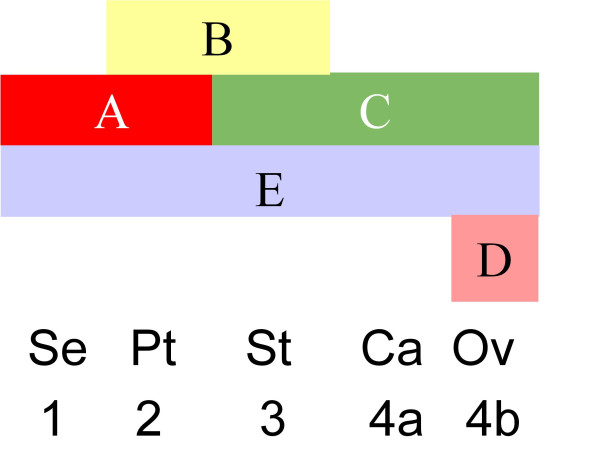
**The ABCDE model for flower organ determination in *Arabidopsis***. The figure has to be read column-wise. E.g., in the sepal-whorl 1, genes A and E are dominantly expressed.

The ABC model links spatial gene expression patterns to phenotypes. Figure 1 shows the expression domains of the five gene classes corresponding to the four types of floral organs: sepals, petals, stamens and carpels (including ovules). For clarity, the carpels and ovules will here be regarded as one organ type. The figure illustrates that, for example, sepal identity is determined by (high) expression of A and E type genes, and this normally occurs in whorl 1, the most outer whorl of the floral meristem. In *Arabidopsis*, the five gene classes in the ABCDE model comprise several redundant genes. The A type genes are represented by *APETALA1 (AP1) *and *APETALA2 (AP2)*, the B type by *AP3 *and *PI*, the C type by *AGAMOUS (AG)*, the D type by *SHATTERPROOF1 *and *SHATTERPROOF2 (SHP1, SHP2) *and *SEEDSTICK (STK)*, and the E type by *SEPALLATA1-SEPALLATA4 (SEP1, SEP2, SEP3 *and *SEP4)*. *SEP1-3 *are expressed in whorl 2-4, and *SEP4 *is expressed in all whorls. The A class gene AP2 is exceptional in that it is the only floral organ identity gene that does not belong to the MADS domain transcription factor family. Although many of the ABCDE genes have been studied extensively, the detailed genetic and molecular interactions among the various redundant genes are still not known comprehensively, and for a number of genes sufficiently detailed expression data are lacking. Therefore, as a first model simplification, we only take MADS box genes into account and we assume that (partially) redundant genes have similar dynamic expression patterns and similar interactions, and can therefore be represented by a single gene from each clade. This is a common approach in literature in dealing with redundancy [8,9,11,14]. The following representative genes are selected to represent the five ABCDE functions: *AP1 *(A), *AP3 *(B), *PI *(B), *AG *(C), *SHP1 *(D) and *SEP *(E). Here, *SEP *denotes *SEP3 *in whorl 2-4, and *SEP1-SEP4 *in whorl 1-4.

## Methods

### Network properties

**Figure 2:**
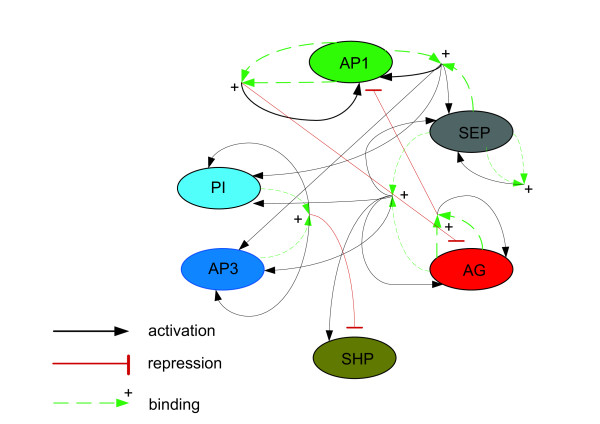
Graphical representation of the interactions in Table 1.

Dimers are known to play an important role in the dynamics of the MADS protein network and represent the minimal structural unit essential for DNA binding [27,29]. We therefore explicitly include dimers in our model and consider their regulatory functions as known or suggested by indirect genetic evidence (Table 1) [25,34,26]. Figure 2 shows the network interactions graphically. Table 2 gives the expected protein expression patterns in the different whorls from day 2 to 5 of meristem development. Before day 2, no initiation of floral organ primordia and differentiation of the different floral organs takes place, and the genes are expressed uniformly over the floral meristem.

**Table 1:** Dimers that are supposed to be repressing (right column) or activating (middle column) the transcription of the individual MADS domain proteins (left column).

Protein	Activator	Repressor
AP1	[AP1 SEP]	[AG AG]
AP3	[AP1 SEP], [AG SEP], [AP3 PI]	
PI	[AP1 SEP], [AG SEP], [AP3 PI]	
AG	[AG SEP], [AG AG]	[AP1 AP1]
SHP	[AG SEP]	[PI AP3]
SEP	[AG SEP], [AP1 SEP], [SEP SEP]	

**Table 2:** Expressed MADS box genes for each floral whorl from day 2 to 5 of meristem development. Day 2 means the end of the second day. SEP (E) is abundant everywhere at all times.

Whorl day	2	3	4	5
1 (sepals)	AP1	AP1	AP1	AP1
2 (petals)	AP1,AP3,PI	AP1,AP3,PI	AP1,AP3,PI	AP1,AP3,PI
3 (stamens)	AP3,PI,AG	AP3,PI,AG	AP3,PI,AG	AP3,PI,AG
4 (carpels)	AG	AG	AG,SHP	AG,SHP

### Dynamical model

To model the dynamical behavior of the MADS box genes, we write down the governing differential equations, which are based on the following set of assumptions: (a) Intercellular diffusion of MADS box proteins is, on average, small. With diffusion ignored, ODE's instead of partial differential equations (PDE's) can be used. The different whorls are modeled with whorl-specific triggering mechanisms, representing the timed initial activation of the MADS domain proteins by non-MADS factors, such as *LEAFY (LFY)*, and *WUSCHEL (WUS) *[22,23]. (b) After activation, MADS box gene transcription is regulated by auto-regulatory mechanisms in which protein dimers play an essential role. (c) Transcription only occurs when one or more activation sites on the DNA are occupied, and simultaneously all repression sites are empty. This assumption is commonly made in modeling of gene regulatory networks [35]. (d) The delay effect of translation is neglected, i.e. transcription immediately leads to protein formation. (e) Dimer decay into non-functional components is small compared to decay into functional monomers [36,37] and is therefore ignored. (f) During the first five days of meristem development, the average cell size remains approximately the same [38].

The dynamics of the dimer concentrations consists of the association rate of monomers into dimers, minus the dissociation rate of dimers into monomers. Denoting by *x*_*i *_the concentration of monomer *i *and by [*x*_*i*_*x*_*j*_] the concentration of the dimer of proteins *i *and *j*, we have the following balance equation(1)

The proteins represented by the variable *x*_*i *_are listed in Table 3.

**Table 3:** Notation for the variables that represent the MADS proteins.

variable	MADS
*x*_1_	AP1
*x*_2_	AP3
*x*_3_	PI
*x*_4_	AG
*x*_5_	SHP
*x*_6_	SEP

The dynamics of monomer concentrations is more complex. It depends not only on the dimer association/dissociation rates, but also on transcriptional regulation and decay into nonfunctional elements. Transcriptional regulation is modeled by Michaelis-Menten functions, in which *β *represents the maximum transcription rate, *Km *the halfmaximal activation or repression, and *dc *the decay rate. As additional elements to the model we include two triggers, *p*_2_(*t, w*) and *p*_4_(*t, w*), which govern the expression of genes AP3 and AG, respectively, at time *t *in whorl *w*. The expression of AP3 is regulated by the genes *LFY *and *UNUSUAL FLORAL ORGANS (UFO) *[39], and the expression of AG is regulated by the genes *WUS *and *LFY *[40,41]. Since these terms are the only time-and whorl-dependent components in the model, they are responsible for cell differentiation. The triggers are essential to drive the network into four different steady states, where each one corresponds to a different organ identity. The biological mechanism that is responsible for the trigger is not modeled here. Quantitative information on the amount of protein generated by the triggers is not available, but their timing is known. Because autoregulatory loops can maintain the expression of the MADS box genes after induction, the duration of the triggers is set to one day. The triggers *p *are thus assumed to take on a nonzero constant value between day 1 and 2 only, and otherwise are set to zero. Altogether, this gives the following model for the monomer dynamics:(2)

Here, the first fractions on the right hand sides denote activation or repression by Michaelis-Menten kinetics, followed by a decay term. The last terms denote the rates of dimerization, which, when positive, act to decrease monomer concentrations. Per whorl, the network dynamics is governed by equation sets (1)-(2), which involves 13 equations, 13 variables, and 51 parameters. To enable parameter estimation, we reformulate the model entirely in terms of monomer concentrations. Another advantage to this is that elimination of the dimer variables and dynamics considerably simplifies the analysis, since it reduces the number of equations and variables to 6. This makes the search algorithm easier to implement and faster. Reformulation is done, first, by applying a time scale decomposition to (1). For a comprehensive background to this technique, see e.g. [42], p.168. The time constant is *days*, which implies that on a scale of days, the dynamics of dimer formation is very fast. This justifies the use of a quasi-steady state approach, in which the dimer concentrations are fully determined by the instantaneous monomer concentrations. This effectively comes down to setting the time derivatives in (1) to zero. By doing so, the dimer equations (1) take the form(3)

and these are inserted into (2), using the chain rule:(4)

If these expressions are substituted in (2), we obtain a system of 6 equations with 6 variables:(5)

Here,  The ordering of γ_*j *_is discussed below. We are aware that these equations now attain a form that is quite unusual for ODE's, since the derivatives are also present in the right hand sides. We explain in the following section why this form is still useful in the context of parameter estimation.

### Parameter estimation

The 37 parameters in (5) need to be estimated from measured time series. This estimation is done in three steps. First, the allowed parameter ranges are defined. Second, a data set is presented and converted into the desired whorl-specific form. Third, the network and data set are decoupled to allow a successful identification procedure.

#### Parameter values

Because the number of parameters is relatively large compared to the number of data points, straightforward estimation might be problematic. Hence, we choose to treat different parameters on a somewhat different footing, depending on available biological knowledge about allowed parameter ranges.

In [43] values are given for *β *in the range of [3, 41] *nMmin*^-1 ^(*α*, Table 3). We therefore take [1, 50] *nMmin*^-1 ^as a reasonable range for *β*. In the same table in [43], *Km *(there named THR) is given in the range of [10^2^, 10^3^] *nM*, and in [44] values used for *Km *(there named )) are in the range of [10, 10^2^] *nM*. Therefore, a reasonable range for *β *is [1, 50] *nMmin*^-1^. A range for decay of dc ∈ [10^-3^, 10^-1^] *min*^-1 ^is given in [45]. Ranges for association and dissociation constants are *K*_*on *_∈ [10^-3^, 1] nM^-1^*min*^-1 ^and *K*_*off *_∈ [10^-3^, 10] *min*^-1^[45]. The relative interaction strengths between dimerising proteins are based on expert knowledge:(6)

where {*x*_*i*_*x*_*j*_} denotes the value of *K*_*on*_/*K*_*off *_corresponding to the dimer [*x*_*i*_*x*_*j*_]. Based on this information, we fix the values of these parameters at *K*_*off *_= 1, γ_1 _= 1, γ_2 _= 10^-1^, γ_3 _= 10^-1^, γ_4 _= 10^-2^, γ_5 _= 10^-2^, γ_6 _= 5.10^-3^, and γ_7 _= 5.10^-3^*nM*^-1^.

### Data manipulation

Data from [21] contain the mRNA signal intensities of the six genes included in our model, at the first five consecutive days of floral meristem development. AP1, which is normally activated by *LFY *and *FLOWERING LOCUS T (FT) *[39,46], is induced here artificially at time point 0. The measured *SEP3 *concentrations are expected to be representative for the concentrations of *SEP1-SEP4 *in whorl 2-4, and for *SEP4 *in whorl 1. The intensities are averages over the whole meristem. Since we need whorl-specific data, the data set is transformed from average intensities to whorl specific protein concentrations in five steps.

1. The data set is scaled uniformly from mRNA intensity to protein concentration, such that the average concentration is 10^3^*nM *(in [45] a range of 10^2^-10^4 ^*nM *is mentioned for transcription factors in eukaryotic cells). Here we implicitly assume that the microarray signal intensities have a linear correspondence to the protein concentrations. This is based on the observation that spatial mRNA expression levels and protein levels correspond well to each other for some of the ABC class MADS transcription factors [47].

2. If in a whorl a gene is not expressed, the protein concentration is set to 1% of the value of a protein that is expressed. This is based on visual interpretation of confocal images from [47].

3. The gene expressions per time point and whorl are based on [20] and are given in Table 2.

4. The relative whorl sizes are obtained from confocal images from [47]. From day 2 to day 5, organ identity comes into play. The shapes and relative sizes stay approximately the same between day 2 and 5. At the end of day 2, the sepals have a volume of 1.1·10^4^*μm*^3^, the petals of 2.7·10^4^*μm*^3^, the stamens of 2.9·10^4^*μm*^3^, and the carpels of 1.1·10^4^*μm*^3^.

5. The mass balance for the concentration of protein *i *is(7)

Here, *w *runs over the whorls,  is the average concentration of protein *i *from the data set,  the concentration of protein *i *in organ *w*, and *V*^*w*^(t) the organ volume. Further,(8)

with  the percentage of expression (1% when there is no expression, 100% when the gene is expressed), and *α*_*i*_(*t*) a scaling factor. To determine *α*_*i*_(*t*), equation (8) is inserted into (7), which yields the expression(9)

#### Network decoupling

**Figure 3:**
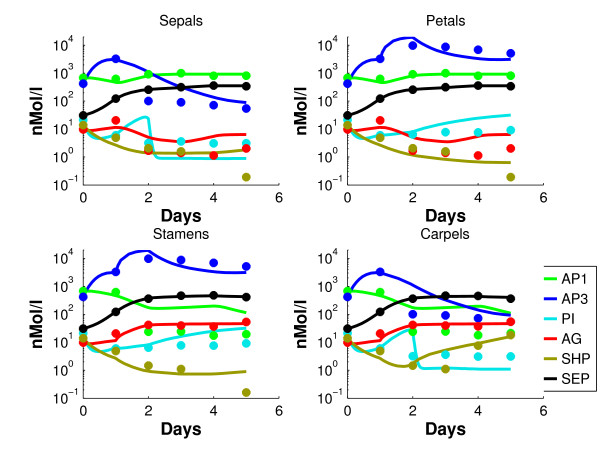
**Simulated dynamics of the decoupled model (5) (solid lines) of the monomers, together with the data points for the four organs**.

**Figure 4:**
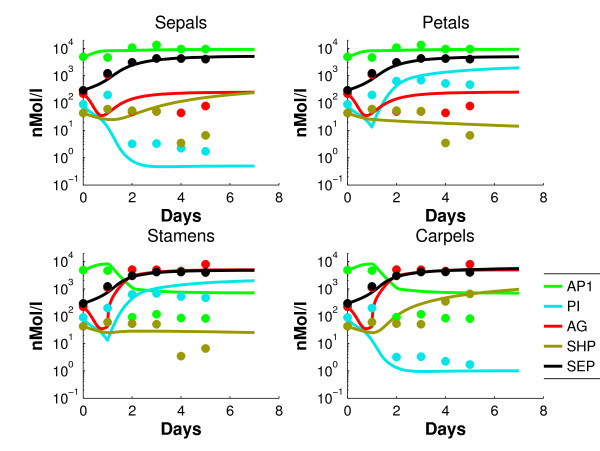
**Simulated dynamics of the coupled network (1)-(2) (solid lines) of the concentrations of proteins that are part of dimer complexes, together with the data points for the four organs**.

With the *γ*_*i *_values given, the system of ODE's (5) still contains 37 parameters that need to be estimated. This puts a high computational demand on the search algorithm, which we propose to alleviate by using a decoupling procedure [48-51]. This approach is based on a simple, highly effective idea. Let us explain this for the decoupling of equation 5(a), which has the form:(10)

with *par *the set of parameters in this equation. For concentrations *x*_2, _.., *x*_6 _and  we take the data and interpolate them with a forward Euler method. This basic interpolation scheme is straightforward and does not introduce substantial interpolation errors. In the end the decoupled network of monomers has the same quality of data fit as the coupled dimer network, Figures 3 and 4.

The resulting functions *x*_2_(*t*), ..., *x*_6_(*t*) and  are substituted in (10) so that in the resulting equation *x*_1 _is the only variable. This equation is integrated and by fitting the calculated values of *x*_1 _to the data for *x*_1_, the parameters *par *are found. This procedure thus leads to estimates for the subset of parameters in (10). Similarly, the other parameters in 5(b)-5(f) are estimated by decoupling the equation under consideration from the others. Note that this reduction method is applicable thanks to the fact that no parameter appears in more than one equation.

The measured concentrations are those of the total amount of *x*_*j *_, both in monomer and dimer form, which are denoted by . To calculate the monomer concentrations from the data, we use mass balances. From the dimers listed in section Network properties, we find that(11)

The quasi-steady state equations (3) are inserted into (11), which yields a set of nonlinear equations in the monomer variables. Finally, the monomer concentrations are estimated by a nonlinear search algorithm for each time point and whorl in the data set.

#### Parameter identification

The identification criterion is to minimize the least squares error(12)

by optimization over the parameter vector *a*, that consists of the unknown parameters in (5). Here  are the data points in whorl *w *for protein *j *at time *t*_*i *_(in days), and *x*_*j*_,_*w*_(*t*_*i*_, *a*) the concentrations that are predicted by our model for some choice of parameter vector *a*. The optimization of *a *is carried out by the Matlab routine "lsqnonlin", which is a gradient-based search method [52,53]. The initial concentrations (at *i *= 0) are taken equal to the data points. For the integration we used the Matlab ode23 Dormand-Prince algorithm [54].

As for the choice of initial values, we investigated several strategies and found that the following choice led to the fastest convergence of the search algorithm. The initial parameter values *K*_*m *_were chosen such that for typical concentration values the Michaelis-Menten functions attain their maximal slopes and therefore are highly sensitive for parameter variations.

The search space for *a *is confined as much as possible to the parameter ranges that are listed in section Parameter values. In Additional file 1 a robustness analysis is presented which aimed to assess whether the optimal parameter values that are retrieved are sensitive to the choice of the γ's. It turned out that the local minima are robust against variation in any γ by at least a factor two.

## Results

### Parameter estimation

The estimated parameter values are listed in Table 4.

**Table 4:** Identified parameters for model (5). *β*_*i,j *_is the maximal transcription rate in *nM day*^-1 ^for the *j *th Michaelis-Menten function of gene *i*, *Km*_*i,j *_the corresponding half-maximal activation in *nM*, *d*_*i *_the decay in *day*^-1^, and *P*_*i *_the trigger in *nM day*^-1^.

*β*_1,1_	6.6e4	*Km*_1,1 _	10	*Km*_6,1 _	5.7e2
*β*_2,1 _	3.3e4	*Km*_1,2 _	3.7e2	*Km*_6,2 _	20
*β*_2,2 _	1.2e2	*Km*_2,1 _	6.1e2	*Km*_6,3 _	47
*β*_2,3 _	1.2e2	*Km*_2,2 _	1.1e3	*dc*_1 _	71
*β*_3,1 _	1.5e3	*Km*_2,3 _	1.1e2	*dc*_2 _	3
*β*_3,2 _	38	*Km*_3,1 _	3.1e2	*dc*_3 _	48
*β*_3,3 _	38	*Km*_3,2 _	6.3e2	*dc*_4 _	5e2
*β*_4,1 _	8.8e3	*Km*_3,3 _	63	*dc*_5 _	4
*β*_4,2 _	1.5e4	*Km*_4,1 _	10	*dc*_6 _	16
*β*_5,1 _	4.1e2	*Km*_4,2 _	1e2	*P*_2 _	4.5e4
*β*_6,1 _	2.3e3	*Km*_4,3 _	1.1e3	*P*_4 _	3.4e3
*β*_6,2 _	44	*Km*_5,1 _	1e3		
*β*_6,3 _	6e3	*Km*_5,2 _	10		

According to section Parameter values, the maximal transcription rate, which is the sum of the *β*'s in each equation, should lie in the range of [1.4·10^3^, 7·10^4^] *nM/day*. All the (sums of the) *β*'s are within this range, except for *β*_5,1_, which is a factor 3.5 lower. The values of *K *are all within the range of [10, 10^3^] *nM*^-1^, with the exception of *Km*_2,2 _and *Km*_4,3_, which are only slightly higher. All decay rates are within the range of [1, 1.5·10^2^] day^-1^, except *dc*_4_, which is somewhat higher.

Figure 3 shows the simulated *decoupled *dynamics of the *monomer *proteins in the identified model (5), together with the data points.

**Figure 5:**
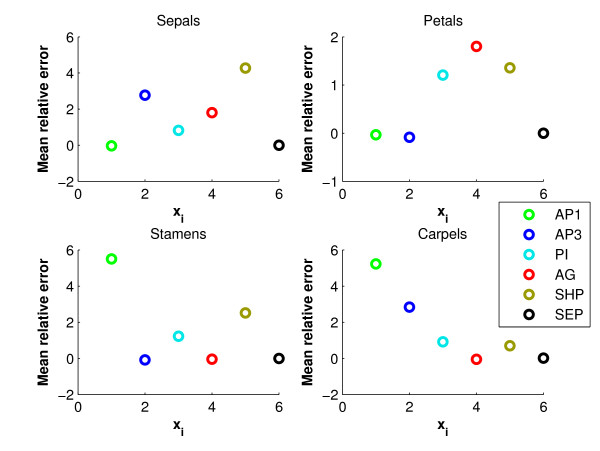
**Mean relative error between simulation and experiment as defined in equation (13)**. The horizontal axis corresponds to the variables [*x*_1, _.., *x*_6_].

For convenience, highly expressed genes will be called "on" and lowly expressed genes will be called "off". AP1 has a good fit in the sepals and petals, where the gene is on. In stamens and carpels, AP1 is a factor 10 too high from day 2-5, but still a factor 5 lower than the on-level. The contributions of the triggers of AP3 (petals and stamens) and AG (stamens and carpels) between day 1 and 2 are clearly distinguishable. PI shows an overshoot between day 1 and 2 in the sepals and carpels. It has to be mentioned that the log-scale visually magnifies errors in the low concentration regime. Since the assumption that the off-genes have 1% of the concentration of the on-genes is an estimation that induces unavoidable errors in the low regimes, these are considered less important. A parameter set is found that fits the monomeric concentration data quite reasonably. It is well known [30] that cell identity is determined by complexes of the ABCDE genes, instead of only by the monomers. Therefore, the monomer concentrations of the model (1)-(2) are converted into *dimer *concentrations (i.e. concentrations of proteins that are part of a dimer), using (3) and the mass balance (11). The data set is converted similarly. Figure 4 shows the profiles of the concentrations of proteins that are part of *dimer *complexes in the resulting *coupled *network. Since AP3 and PI only form the dimer [AP3 PI], their concentrations are equal. Therefore AP3 is omitted in the dimer concentration plots from now on. The simulated concentrations remain more or less consistent after day 5, which one would expect. It could be expected that the fit of the coupled network in Figure 4 is less accurate than the fit of the decoupled equations in Figure 3. Conversion into dimer concentrations could result in further inaccuracies, since the parameters are fitted on monomeric data. However, comparison with Figure 3 shows that there are not many substantial differences between the coupled and decoupled network. Figure 5 shows for each whorl and each gene the mean relative error , which is defined as follows(13)

where *x *is the simulated and *x*_*d *_the measured gene expression. In (13) we take on purpose not the squares of the differences, since epsilon should measure whether *x*_*d *_is systematically over-or underestimated over five days. Moreover, the differences *x-x*_*d *_have been normalized by *x*_*d *_in order to make a comparison between concentrations of different orders of magnitude possible. Based on Figures 4 and 5 we make the following observations:

From a qualitative point of view, the fitted model reproduces the expression data very reasonably. This implies that the topology of the model apparently includes the most relevant interactions. AP1 has a reasonable fit in the sepals and petals, where it is on. In the stamens and carpels it is too high after day 2, by a factor 8. However, the AP1 values are still a factor 12 below the on-level. AG has a good fit for the on-levels in stamens and carpels, and in the sepals and petals it compromises between the high values at day 1, and the low levels later on. SHP is on only in the carpels after day 3, and here the fit is accurate. In the other organs there is a compromise between the high levels during day 0-3, and the low levels at days 4-5. SEP has an accurate fit for all organs.

### Model validation by mutants

A powerful method to test the validity of our model is to compare the expression measurements in plants with mutations in particular MADS transcription factors with the MADS protein concentrations predicted by the model. The mutations can be simulated by either setting the initial concentration and production of protein from a knocked out gene equal to zero, or by fixing the concentration to a high level if the gene is ectopically expressed. We applied this test for five known mutants in *Arabidopsis*. For four mutants, the model predicted the correct phenotypes very well. One mutant was only partly predicted correctly. This confirms the predictive power with respect to genetic mutations.

**Figure 6:**
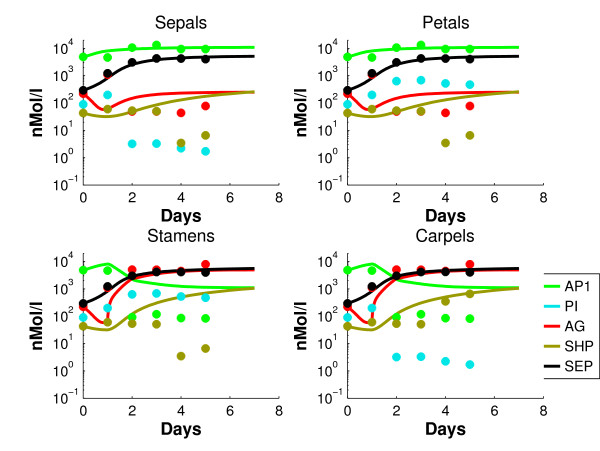
**Simulated dynamics for the AP3 = 0 mutant: the second whorls grow sepals, and the third whorl grows carpels**. The data points denote the wild-type expression levels, and they are shown to compare the mutant dynamics with.

In the first mutant, AP3 is missing. According to [55], the second whorl grows sepals, and the third whorl grows carpels. Figure 6 shows that our model predictions are in agreement with these phenotypic alterations. Indeed, we observe that the expressions in the second whorl agree with the first, and therefore they develop the same organs. The same applies to the third and fourth whorl. The expression levels of the B-gene *PI *become so small that they are not visible in Figure 6, due to the logarithmic scale. In the stamens the B-genes are off, and since in this model SHP is repressed by the AP3-PI dimer, the third whorl develops the same SHP levels as the fourth whorl. This repression assumption has never been proven, but is now supported by the outcomes of this simulation. The results of the remaining mutants are mentioned in brief hereafter. A more elaborate discussion of the simulation results is presented in Additional file 2.

In the second mutant, PI is missing. The same phenotype occurs as with AP3 missing [1], and this is in agreement with the model predictions. In the third mutant, AP3 is ectopically expressed. According to the model prediction, the fourth whorl organs have stamen expression, and the first whorl organs have petal identities. However, according to [36], the fourth whorl organs develop as stamens, but there is no change in the identities of the first whorl organs. The model prediction is thus only partially correct. In the fourth mutant, AG is ectopically expressed. According to the model prediction, the first whorl grows carpels, and the second whorl grows stamens. This is in agreement with the findings in [56]. In the fifth mutant, AG is absent in all whorls. According to [57], AG is involved in terminating the meristem activity in the flower after the formation of the fourth whorl carpels. Without AG, only sepals and petals are formed and the inner part of the meristem starts to develop new floral buds that reiterate the formation of sepals and petals ad infinitum. The AG stop mechanism is not modeled here, so in our simulations only 4 whorls of floral organs develop. Without the C gene, the A gene is not repressed in any floral organ, and will therefore be expressed in the whole floral meristem throughout flower development. According to the ABC model in Figure 1, whorls 1-4 will have sepals-petals-petals-sepals identity, respectively, and this is exactly what our model predicts.

### Model predictions

Recently we developed a bioinformatics method to predict specific sites in protein sequences where mutagenesis changes protein interaction specificity [58]. For various MADS proteins, interaction specificity was changed experimentally, and validated using yeast-two-hybrid experiments. This is essentially a new type of mutation experiment, for which the phenotypic effects have not yet been investigated *in planta*. This kind of experiments can however easily be mimicked with the present model. We present results of this type of simulations to allow for the possibility of comparing them with future experiments. As a model prediction, the interaction strength of each dimer γ_*i *_is set to zero, one at a time. This comes down to removing one specific dimer from the network. Table 5 shows the predicted phenotypic alterations.

**Table 5:** Predicted organ mutations for each removed dimer binding affinity.

Removed dimer	Phenotypic alteration
[*AG AG*]	stamens → petals, carpels → sepals
[*AG SEP*]	stamens → petals, carpels → sepals
[*SHP SEP*]	carpels: no ovules
[*AP*1 *SEP*]	sepals → carpels, petals → stamens
[*AP*3 *PI*]	petals → sepals, stamens → carpels
[*AP*1 *AP*1]	sepals → carpels, petals → stamens
[*SEP SEP*]	no development of floral organs

It turns out that all dimer mutations show very clear organ conversions. It is interesting to see that all dimers play an important role in organ specification, even the dimers with very low association rates. Experimental verification of these predictions can provide a valuable tool for a more accurate determination of parameter values, as well as model structure, without the need for costly time series of expression data.

## Discussion

The mutant simulation experiments show realistic results, and only one mutant (out of five) could not fully be reproduced. This indicates that we developed a very useful model, despite that it was based on only a limited amount of quantitative data. All seven dimer mutants are predicted to have phenotypic effects. In five out of seven mutations, double organ conversions occur, and for one mutation no floral organs formed at all, as expected in view of the known importance of the SEP proteins for determining floral organ identity.

These predictions, however, have to be interpreted with caution. The simulated dynamics depends on parameter values that have an uncertainty intrinsic to the sparse and uncertain data set used. Hence, accurate quantitative predictions are yet out of reach. Nevertheless, on a qualitative scale the mutant predictions suggest that the functional loss of each dimer leads to phenotypic alteration, and that therefore each dimer plays an essential role in the regulatory network. At this moment, our group is performing confocal image analysis of MADS protein expression patterns and levels to obtain a high-quality and quantitative protein expression data set. We are also investigating the existence of additional genetic interactions. We expect that this will result in a complete network with accurate parameter values, that opens the door for testing other candidate model structures. For example, an additional hypothesis on the mode of action of the MADS proteins in the gene regulatory network that controls floral organ specification is proposed in [31] and states that the MADS proteins act in quaternary complexes, consisting of two dimers. Over the last few years more and more evidence has become available showing the formation [59,30,32] and DNA binding capacity of such complexes [59,60].

## Conclusions

We proposed an ODE model for the dynamics of six genes that regulate floral organ identity in *Arabidopsis*. The model describes transcription regulation, mass balance, dimer formation, decay and cellfate determining trigger mechanisms. The parameters are estimated by an identification method that comprised a network decoupling method. The data set used consists of microarray intensities from the literature. The resulting model is validated by predicting the phenotypes of five mutants known from literature. Also, some new model predictions are made for an *in vitro *type of mutants, in which the formation of specific dimers is artificially repressed. Thanks to its well-defined mathematical and biological foundation, the model can be easily extended with additional biological knowledge. The model structure allows a decoupling procedure that seems to be a promising identification technique. Its application is apparently generic for ODE models of gene regulatory networks.

Experimental verification of the dimer mutants could provide a valuable tool for confirmation, falsification and/or model refinement. In systems biology, the cyclic interaction between 'wet-lab' experiments and 'dry-lab' modeling plays a defining role. In this light we have presented here several mutants for which our model predicts a phenotype, in the hope to inspire biologists to test and study these special cases experimentally.

## Authors' contributions

SM: Mathematical model analysis, simulation studies and software development, wrote the article. AD, RI, KK, GA and RH: Model development, data analysis and manipulation, scientific background. MG and JM: Mathematical model analysis and co-wrote the article. All authors read and approved the manuscript.

## Supplementary Material

Additional file 1 **Sensitivity analysis of the optimal parameters with respect to binding affinities**. Click here for file

Additional file 2 **Discussion on the simulation results of the mutants**. Click here for file

## References

[B1] Coen E Meyerowitz E The war of the whorls: genetic interactions controlling flower development Nature 1991 353 31 37 10.1038/353031a0 1715520

[B2] Ferrario S Immink R Angenent G Conservation and diversity in flower land Current opinion in plant biology 2004 7 84 91 10.1016/j.pbi.2003.11.003 14732446

[B3] Causier B Schwarz-Sommer Z Davies B Floral organ identity: 20 years of ABCs Seminars in Cell & Developmental Biology 2010 21 73 79 http://www.sciencedirect.com/science/article/B6WX0-4XKBYX0-2/2/72df2c458605237d76c5635658302624 [Tumor-Stroma Interactions; Flower Development]. 10.1016/j.semcdb.2009.10.005 19883777

[B4] Colombo L Franken J Koetje E vanWent J Dons HJM Angenent GC van Tunen AJ The Petunia MADS Box Gene FBP11 Determines Ovule Identity The Plant Cell 1995 7 11 1859 1868 10.2307/3870193 8535139 PMC161044

[B5] Pelaz S Ditta GS Baumann E Wisman E Yanofsky MF B and C floral organ identity functions require SEPALLATA MADS-box genes Nature 2000 405 200 203 10.1038/35012103 10821278

[B6] Favaro R Pinyopich A Battaglia R Kooiker M Borghi L Ditta G Yanofsky MF Kater MM Colombo L MADS-box protein complexes control carpel and ovule development in Arabidopsis Plant Cell 2003 15 2603 2611 10.1105/tpc.015123 14555696 PMC280564

[B7] Krizek BA Fletcher JC Molecular mechanisms of flower development: an armchair guide Nat Rev Genet 2005 6 688 698 10.1038/nrg1675 16151374

[B8] Espinosa-Soto C Padilla-Longoria P Buylla E A gene regulatory network model for cell-fate determination during *Arabidopsis thaliana *flower development that is robust and recovers experimental gene expression profiles The Plant Cell 2004 16 2923 2939 10.1105/tpc.104.021725 15486106 PMC527189

[B9] Mendoza L Thieffry D Alvarez-Buylla ER Genetic control of flower morphogenesis in Arabidopsis thaliana: a logical analysis Bioinformatics 1999 15 593 606 10.1093/bioinformatics/15.7.593 10487867

[B10] Alvarez-Buylla ER Azpeitia E Barrio R Bentez M Padilla-Longoria P From ABC genes to regulatory networks, epigenetic landscapes and flower morphogenesis: Making biological sense of theoretical approaches Seminars in Cell & Developmental Biology 2010 21 108 117 http://www.sciencedirect.com/science/article/B6WX0-4XP381V-6/2/5e0534192b4f9a12b9f6a54f2cb94735 [Tumor-Stroma Interactions; Flower Development]. 10.1016/j.semcdb.2009.11.010 19922810

[B11] Alvarez-Buylla ER Chaos A Aldana M Bentez M Cortes-Poza Y Espinosa-Soto C Hartasnchez DA Lotto RB Malkin D Escalera Santos GJ Padilla-Longoria P Floral morphogenesis: stochastic explorations of a gene network epigenetic landscape PLoS ONE 2008 3 e3626 10.1371/journal.pone.0003626 18978941 PMC2572848

[B12] Karlebach G Shamir R Modelling and analysis of gene regulatory networks Nature 2008 9 770 780 10.1038/nrm2503 18797474

[B13] Pahle J Biochemical simulations: stochastic, approximate stochastic and hybrid approaches Brief Bioinformatics 2009 10 53 64 10.1093/bib/bbn050 19151097 PMC2638628

[B14] Lenser T Theissen G Dittrich P Developmental robustness by obligate interaction of class B floral homeotic genes and proteins PLoS Comput Biol 2009 5 e1000264 10.1371/journal.pcbi.1000264 19148269 PMC2612583

[B15] Agrawal S Archer C Schaffer DV Computational models of the Notch network elucidate mechanisms of context-dependent signaling PLoS Comput Biol 2009 5 e1000390 10.1371/journal.pcbi.1000390 19468305 PMC2680760

[B16] Cinquin O Repressor dimerization in the zebrafish somitogenesis clock PLoS Comput Biol 2007 3 e32 10.1371/journal.pcbi.0030032 17305423 PMC1797823

[B17] Porreca R Drulhe S de Jong H Ferrari-Trecate G Structural identification of piecewise-linear models of genetic regulatory networks J Comput Biol 2008 15 1365 1380 10.1089/cmb.2008.0109 19040369

[B18] Farcot E Gouzé JL A mathematical framework for the control of piecewise-affine models of gene networks Automatica 2008 44 9 2326 2332 http://www-sop.inria.fr/virtualplants/Publications/2008/FG08 10.1016/j.automatica.2007.12.019

[B19] Gennemark P Wedelin D Benchmarks for identification of ordinary differential equations from time series data Bioinformatics 2009 25 6 780 786 10.1093/bioinformatics/btp050 19176548 PMC2654804

[B20] Schmid M Davison TS Henz SR Pape UJ Demar M Vingron M Schlkopf B Weigel D Lohmann JU A gene expression map of Arabidopsis thaliana develop-ment Nat Genet 2005 37 501 506 10.1038/ng1543 15806101

[B21] Wellmer F Alves-Ferreira M Dubois A Riechmann J Meyerowitz E Genome-wide analysis of gene expression during early arabidopsis flower development PLoS Genetics 2006 2 7 1012 1024 10.1371/journal.pgen.0020117 PMC1523247 16789830

[B22] Lohmann JU Weigel D Building beauty: the genetic control of floral patterning Dev Cell 2002 2 135 142 10.1016/S1534-5807(02)00122-3 11832239

[B23] Liu Z Mara C Regulatory mechanisms for floral homeotic gene expression Semin Cell Dev Biol 2010 21 80 86 10.1016/j.semcdb.2009.11.012 19922812

[B24] Kaufmann K Melzer R Theissen G MIKC-type MADS-domain proteins: structural modularity, protein interactions and network evolution in land plants Gene 2005 347 183 198 10.1016/j.gene.2004.12.014 15777618

[B25] de Folter S Angenent GC Trans meets cis in MADS science Trends Plant Sci 2006 11 224 231 10.1016/j.tplants.2006.03.008 16616581

[B26] Kaufmann K Muio JM Jauregui R Airoldi CA Smaczniak C Krajewski P Angenent GC Target genes of the MADS transcription factor SEPALLATA3: integration of developmental and hormonal pathways in the Arabidopsis flower PLoS Biol 2009 7 e1000090 10.1371/journal.pbio.1000090 19385720 PMC2671559

[B27] Riechmann JL Krizek BA Meyerowitz EM Dimerization specificity of Arabidopsis MADS domain homeotic proteins APETALA1, APETALA3, PISTILLATA, and AGAMOUS Proc Natl Acad Sci USA 1996 93 4793 4798 10.1073/pnas.93.10.4793 8643482 PMC39358

[B28] Sablowski R Genes and functions controlled by floral organ identity genes Semin Cell Dev Biol 2010 21 94 99 10.1016/j.semcdb.2009.08.008 19733677

[B29] Pellegrini L Tan S Richmond TJ Structure of serum response factor core bound to DNA Nature 1995 376 490 498 10.1038/376490a0 7637780

[B30] Honma T Goto K Complexes of MADS-box proteins are sufficient to convert leaves into floral organs Nature 2001 409 525 529 10.1038/35054083 11206550

[B31] Theissen G Saedler H Floral quartets Nature 2001 49 469 471 10.1038/35054172 11206529

[B32] Immink RG Tonaco IA de Folter S Shchennikova A van Dijk AD Busscher-Lange J Borst JW Angenent GC SEPALLATA3: the 'glue' for MADS box transcription factor complex formation Genome Biol 2009 10 R24 10.1186/gb-2009-10-2-r24 19243611 PMC2688274

[B33] Immink RG Kaufmann K Angenent GC The 'ABC' of MADS domain protein behaviour and interactions Semin Cell Dev Biol 2010 21 87 93 10.1016/j.semcdb.2009.10.004 19883778

[B34] Gomez-Mena C de Folter S Costa MM Angenent GC Sablowski R Transcriptional program controlled by the floral homeotic gene AGAMOUS during early organogenesis Development 2005 132 429 438 10.1242/dev.01600 15634696

[B35] Alon U An introduction to systems biology. Design principles of biological circuits 2006 Chapman & Hall/CRC

[B36] Jack T Fox GL Meyerowitz EM Arabidopsis homeotic gene APETALA3 ectopic expression: transcriptional and posttranscriptional regulation determine floral organ identity Cell 1994 76 703 716 10.1016/0092-8674(94)90509-6 7907276

[B37] Zachgo S Silva EdeA Motte P Trbner W Saedler H Schwarz-Sommer Z Functional analysis of the Antirrhinum floral homeotic DEFICIENS gene in vivo and in vitro by using a temperature-sensitive mutant Development 1995 121 2861 2875 7555713 10.1242/dev.121.9.2861

[B38] Kwiatkowska D Flower primordium formation at the Arabidopsis shoot apex: quantitative analysis of surface geometry and growth J Exp Bot 2006 57 571 580 10.1093/jxb/erj042 16377735

[B39] Parcy F Nilsson O Busch MA Lee I Weigel D A genetic framework for floral patterning Nature 1998 395 561 566 10.1038/26903 9783581

[B40] Lenhard M Bohnert A Jrgens G Laux T Termination of stem cell maintenance in Arabidopsis floral meristems by interactions between WUSCHEL and AGAMOUS Cell 2001 105 805 814 10.1016/S0092-8674(01)00390-7 11440722

[B41] Lohmann JU Hong RL Hobe M Busch MA Parcy F Simon R Weigel D A molecular link between stem cell regulation and floral patterning in Arabidopsis Cell 2001 105 793 803 10.1016/S0092-8674(01)00384-1 11440721

[B42] Klipp E Kowald A Wierling C Lehrach H Systems biology in practice: concepts, implementation and application 2005 Wiley-VCH

[B43] Cavelier G Anastassiou D Data-Based Model and Parameter Evaluation in Dynamic Transcriptional Regulatory Networks Proteins 2004 55 339 350 10.1002/prot.20056 15048826

[B44] Bundschuh R Hayot F Jayaprakash C Fluctuations and Slow Variables in Genetic Networks Biophysical Journal 2003 84 3 1606 1615 10.1016/S0006-3495(03)74970-4 12609864 PMC1302731

[B45] Buchler NE Louis M Molecular titration and ultrasensitivity in regulatory networks Journal of Molecular Biology 2008 384 1106 1119 10.1016/j.jmb.2008.09.079 18938177

[B46] Wigge PA Kim MC Jaeger KE Busch W Schmid M Lohmann JU Weigel D Integration of spatial and temporal information during floral induction in Arabidopsis Science 2005 309 1056 1059 10.1126/science.1114358 16099980

[B47] Urbanus S de Folter S Shchennikova A Kaufmann K Immink R Angenent G In planta localisation patterns of MADS domain proteins during floral development in Arabidopsis thaliana BMC Plant Biology 2009 9 5 19138429 10.1186/1471-2229-9-5 PMC2630930

[B48] Gennemark P Wedelin D Efficient algorithms for ordinary differential equation model identification of biological systems Systems Biology 2007 1 2 120 129 10.1049/iet-syb:20050098 17441553

[B49] Kimura S Ide K Kashihara A Kano M Hatakeyama M Masui R Nakagawa N Yokoyama S Kuramitsu S Konagaya A Inference of S-system models of genetic networks using a cooperative coevolutionary algorithm Bioinformatics 2005 21 1154 1163 10.1093/bioinformatics/bti071 15514004

[B50] Kimura S Hatakeyama M Konagaya A Inference of S-system models of genetic networks from noisy time-series data Chem-Bio Inform J 2004 4 1 14 10.1273/cbij.4.1

[B51] Maki Y Ueda T Okamoto M Uematsu N Inamura Y Eguchi Y Inference of genetic network using the expression profile time course data of mouse P19 cells Genome Inform 2002 13 382 383

[B52] Coleman T Li Y On the Convergence of Reflective Newton Methods for Large-Scale Nonlinear Minimization Subject to Bounds Mathematical Programming 1994 67 2 189 224 10.1007/BF01582221

[B53] Coleman TF Li Y An Interior, Trust Region Approach for Nonlinear Minimization Subject to Bounds SIAM Journal on Optimization 1996 6 418 445 10.1137/0806023

[B54] Dormand J Prince P A family of embedded RungeKutta formulae Journal of Computational Applied Mathematics 1980 6 19 26 10.1016/0771-050X(80)90013-3

[B55] Jack T Brockman LL Meyerowitz EM The homeotic gene APETALA3 of Arabidopsis thaliana encodes a MADS box and is expressed in petals and stamens Cell 1992 68 683 697 10.1016/0092-8674(92)90144-2 1346756

[B56] Mizukami Y Huang H Tudor M Hu Y Ma H Functional domains of the floral regulator AGAMOUS: characterization of the DNA binding domain and analysis of dominant negative mutations Plant Cell 1996 8 831 845 10.1105/tpc.8.5.831 8672883 PMC161142

[B57] Yanofsky MF Ma H Bowman JL Drews GN Feldmann KA Meyerowitz EM The protein encoded by the Arabidopsis homeotic gene agamous resembles transcription factors Nature 1990 346 35 39 10.1038/346035a0 1973265

[B58] van Dijk AD ter Braak CJ Immink RG Angenent GC van Ham RC Predicting and understanding transcription factor interactions based on sequence level determinants of combinatorial control Bioinformatics 2008 24 26 33 10.1093/bioinformatics/btm539 18024974

[B59] Egea-Cortines M Saedler H Sommer H Ternary complex formation between the MADS-box proteins SQUAMOSA, DEFICIENS and GLOBOSA is involved in the control of floral architecture in Antirrhinum majus EMBO J 1999 18 5370 5379 10.1093/emboj/18.19.5370 10508169 PMC1171606

[B60] Melzer R Theissen G Reconstitution of 'floral quartets' in vitro involving class B and class E floral homeotic proteins Nucleic Acids Res 2009 37 2723 2736 10.1093/nar/gkp129 19276203 PMC2677882

