# Supplementary material for: Continuous-time modeling of cell fate determination in Arabidopsis flowers
Source: BMC Syst Biol. 2010 Jul 22;4:101. doi: 10.1186/1752-0509-4-101 (PMC2922098; doi:10.1186/1752-0509-4-101)
Supplement: Additional file 1 — Sensitivity analysis of the optimal parameters with respect to binding affinities. [file 1752-0509-4-101-S1.PDF]

# Additional file 1 — Sensitivity analysis of the optimal parameters with respect to binding affinities

The proposed parameter search algorithm assumes that the binding affinities $\gamma_i$ are fixed. The values of the $\gamma$'s are not known very accurately, and there is the problem of local minima (discussed in section Parameter estimation). To investigate the robustness of the retrieved optimal parameters with respect to the $\gamma$'s, we applied the search algorithm after varying the $\gamma$'s one by one with factors two and five. Tables 1 and 2 show the optimal parameters that have changed after increasing the $\gamma$'s by a factor two and five respectively. The last line shows the mean relative error between model results and experiment. This error is averaged over all whorls and all genes (compare equations (12) and (13) in the main text), and is defined as

$$\overline{\overline{\varepsilon}} = \frac{1}{120} \sum_{w=1}^{4} \sum_{j=1}^{6} \sum_{i=1}^{5} \frac{x_{j,w}(t_i) - \overline{x_{j,w}(t_i)}}{\overline{x_{j,w}(t_i)}}. \tag{1}$$

Inspection of the tables reveals that increasing the $\gamma$'s with a factor two results generally in small parameter changes. The mean relative error also changes little. An increase of factor five causes more parameters to change, and they change more drastically as well. Nevertheless, the mean relative error does not change much most of the times, except when $\gamma_4$ is increased. Decreasing the $\gamma$'s with a factor five gives similar results, but with the highest mean relative error for variation in $\gamma_5$. This indicates that the optimal parameter set and the corresponding data fit has a robustness against variation of the $\gamma$'s of at least a factor two.

| parameter | nominal | $\gamma_1 * 2$ | $\gamma_2 * 2$ | $\gamma_3 * 2$ | $\gamma_4 * 2$ | $\gamma_5 * 2$ | $\gamma_6 * 2$ | $\gamma_7 * 2$ |
|---|---|---|---|---|---|---|---|---|
| B11 | 66344 | 71147 | 58083 | 66518 | 73312 | 66344 | 63898 | 68805 |
| B21 | 33177 | 33187 | 33199 | 33177 | 33259 | 40000 | 33181 | 33190 |
| B23 | 122 | 122 | 122 | 122 | 100 | 122 | 122 | 122 |
| B41 | 8758 | 10458 | 900 | 8773 | 8566 | 8758 | 900 | 9211 |
| B42 | 14944 | 7009 | 2632 | 14928 | 15246 | 14944 | 22619 | 15393 |
| B51 | 409 | 437 | 501 | 374 | 417 | 373 | 407 | 455 |
| B61 | 2319 | 3178 | 1056 | 2314 | 3944 | 2319 | 1555 | 2310 |
| B62 | 44 | 0 | 1162 | 58 | 0 | 44 | 1031 | 470 |
| B63 | 5996 | 5886 | 10559 | 5956 | 3230 | 5996 | 10491 | 8285 |
| K12 | 374 | 202 | 307 | 374 | 400 | 374 | 368 | 390 |
| K23 | 111 | 111 | 111 | 111 | 91 | 111 | 111 | 111 |
| K42 | 100 | 100 | 100 | 100 | 100 | 100 | 113 | 102 |
| K61 | 573 | 577 | 132 | 571 | 423 | 573 | 513 | 656 |
| K62 | 20 | 20 | 38 | 20 | 20 | 20 | 20 | 20 |
| K63 | 47 | 47 | 143 | 46 | 14 | 47 | 175 | 60 |
| d1 | 71 | 76 | 62 | 71 | 81 | 71 | 93 | 72 |
| d4 | 500 | 500 | 64 | 500 | 500 | 500 | 500 | 500 |
| d6 | 16 | 16 | 28 | 16 | 14 | 16 | 24 | 27 |
| p4 | 3369 | 3767 | 3437 | 3370 | 3145 | 3369 | 2883 | 3369 |
| $\overline{\overline{\varepsilon}}$ | 1.6 | 1.6 | 1.6 | 1.7 | 1.9 | 1.7 | 1.4 | 1.5 |

**Table 1.** The optimal parameters that change by increasing $\gamma$ with a factor 2, together with the mean relative error.

| parameter | nominal | $\gamma_1 * 5$ | $\gamma_2 * 5$ | $\gamma_3 * 5$ | $\gamma_4 * 5$ | $\gamma_5 * 5$ | $\gamma_6 * 5$ | $\gamma_7 * 5$ |
|---|---|---|---|---|---|---|---|---|
| B11 | 66344 | 77226 | 48041 | 66664 | 77710 | 66344 | 63248 | 70858 |
| B21 | 33177 | 33201 | 33238 | 33178 | 33347 | 32727 | 33191 | 33213 |
| B23 | 122 | 122 | 122 | 122 | 82 | 122 | 122 | 122 |
| B41 | 8758 | 8693 | 900 | 8783 | 11118 | 8758 | 911 | 9321 |
| B42 | 14944 | 3209 | 900 | 14918 | 12534 | 14944 | 22676 | 16529 |
| B51 | 409 | 500 | 788 | 357 | 161 | 332 | 404 | 554 |
| B61 | 2319 | 4345 | 0 | 2310 | 6742 | 2319 | 3984 | 2106 |
| B62 | 44 | 0 | 1907 | 69 | 10787 | 44 | 8455 | 782 |
| B63 | 5996 | 5864 | 6254 | 5926 | 95323 | 5996 | 104816 | 11879 |
| K12 | 374 | 87 | 213 | 375 | 423 | 374 | 362 | 412 |
| K23 | 111 | 111 | 111 | 111 | 91 | 111 | 111 | 111 |
| K42 | 100 | 100 | 100 | 100 | 100 | 100 | 122 | 100 |
| K43 | 1111 | 1111 | 1111 | 1111 | 1111 | 1111 | 496 | 1111 |
| K51 | 1000 | 1000 | 1000 | 1000 | 115 | 1000 | 1000 | 1000 |
| K52 | 10 | 10 | 10 | 10 | 417 | 10 | 10 | 10 |
| K61 | 573 | 528 | 10 | 567 | 139 | 573 | 76 | 667 |
| K62 | 20 | 20 | 300 | 20 | 465 | 20 | 40 | 20 |
| K63 | 47 | 49 | 52 | 46 | 860 | 47 | 891 | 48 |
| d1 | 71 | 82 | 51 | 71 | 87 | 71 | 139 | 71 |
| d4 | 500 | 500 | 25 | 500 | 500 | 500 | 500 | 500 |
| d5 | 4 | 4 | 4 | 6 | 4 | 4 | 4 | 4 |
| d6 | 16 | 16 | 22 | 16 | 129 | 16 | 145 | 49 |
| p4 | 3369 | 3835 | 3439 | 3370 | 3051 | 3369 | 2913 | 3171 |
| $\overline{\overline{\varepsilon}}$ | 1.6 | 1.6 | 1.9 | 1.8 | 3.9 | 1.9 | 1.4 | 1.3 |

**Table 2.** The optimal parameters that change by increasing $\gamma$ with a factor 5, together with the mean relative error.
