# Supplementary material for: Continuous-time modeling of cell fate determination in Arabidopsis flowers
Source: BMC Syst Biol. 2010 Jul 22;4:101. doi: 10.1186/1752-0509-4-101 (PMC2922098; doi:10.1186/1752-0509-4-101)
Supplement: Additional file 2 — Discussion on the simulation results of the mutants. [file 1752-0509-4-101-S2.PDF]

Additional file 2 —Discussion on the simulation results of the mutants

After the discussion of the first mutant in the main text, we proceed with the simulation results of mutant experiments 2–5. In the second mutant, PI is missing. The same phenotype occurs as with AP3 missing [2], and this is in agreement with the model predictions and the fact that AP3 and PI form are mutually dependent and form an obligate heterodimer. Figure 1 shows the dimer concentrations in the different whorls.

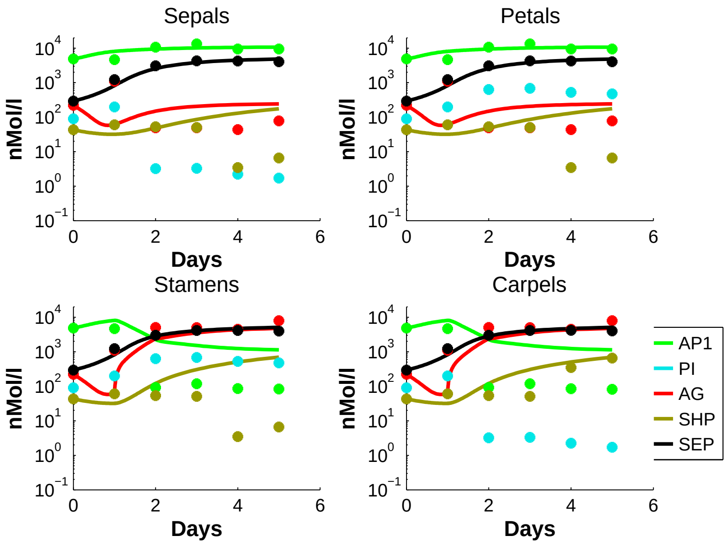

Figure 1. PI=0 mutant: the second whorls grow sepals, and the third whorl grows carpels.

In the second and third whorl, the production term p_2 triggers AP3 for one day, but thereafter it decays quickly. In the ABCDE model, the absence of B genes suggests that the second whorl organs will develop as sepals. SHP is not repressed anymore by the B genes, and as a result it is expressed in the third whorl, which now has carpel-like expression levels. In the third mutant (Figure 2), AP3 is ectopically expressed, i.e. expressed in all organs. The initial concentration of AP3 dimers is set to 5 10^3 nM, which is approximately equal to the on-level. Consequently, AP3 and PI maintain each other at their high levels. It is clear that our model predicts that the fourth whorl organs will have stamen expression, since SHP is suppressed, and that the first whorl organs will have petal identities. However, according to [3] fourth whorl organs develop as stamens, but there is no change in the identities of the first whorl organs. This might be caused by a low PI expression in the first whorl under native conditions, that, together with the suppressing role of AP1, prevents an up-regulation of the two endogenous B genes. Though, in our model PI expression reaches its on level, because of the assumption that in case of no expression 1% protein is present. In the fourth whorl, the suppressing role of AG is apparently not enough to prevent this up-regulation, where as a result the B genes are switched on. With this mutation, more precise parameter values are needed to determine the threshold concentration values (that are inherent to the structures of the Michaelis-Menten kinetics [1]) for up-regulation. Figure 3 shows the simulation results for the fourth mutant. In the first whorl, SHP has the same expression as in the fourth whorl, and AP1 is repressed by AG, which gives the first whorl organs a carpel-like expression pattern. In the second whorl AG is on, and as a consequence AP1 is repressed, which results in a stamen-like expression pattern. In the fifth mutant, AG is absent in all whorls (Figure 4). In the third whorl primordia, AP1 is expressed

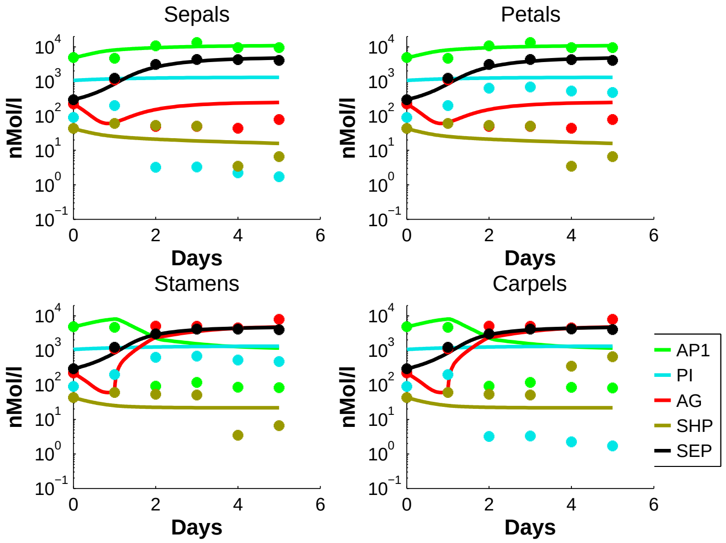

Figure 2. Ectopically expressed AP3 mutant: the fourth whorl organs have stamen expression, and the first whorl organs have petal identities.

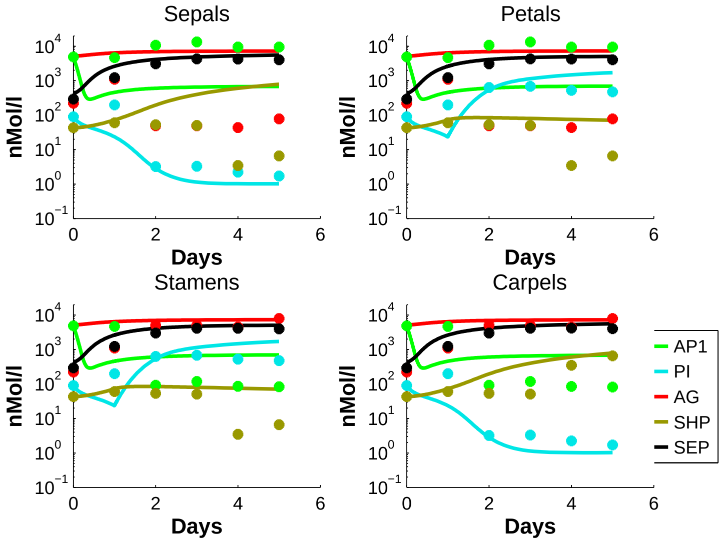

Figure 3. Ectopically expressed AG mutant: the first whorl has carpels, and the second whorl has stamens.

since it is not repressed by AG anymore, and as a consequence the expression pattern matches that of the petals. In the fourth whorl, AP1 is expressed for the same reason, and since SHP is normally promoted by AG, this gene is not expressed. This gives the fourth whorl primordia the same expression levels as the sepals.

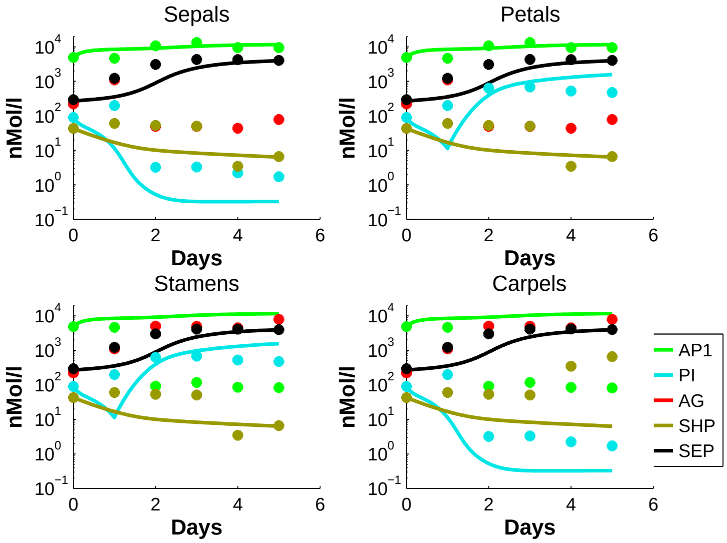

Figure 4. AG=0 mutant: whorls 1–4 have sepals-petals-petals-sepals identity, respectively.

References

1. U. Alon. *An introduction to systems biology. Design principles of biological circuits.* Chapman & Hall/CRC, 2006.

2. E. Coen and E. Meyerowitz. The war of the whorls: genetic interactions controlling flower development. *Nature*, 353:31–37, 1991.

3. T. Jack, G. L. Fox, and E. M. Meyerowitz. Arabidopsis homeotic gene APETALA3 ectopic expression: transcriptional and posttranscriptional regulation determine floral organ identity. *Cell*, 76:703–716, Feb 1994.
